# Imidacloprid Poisoning of Songbirds Following a Drench Application of Trees in a Residential Neighborhood in California, USA

**DOI:** 10.1002/etc.4473

**Published:** 2019-06-26

**Authors:** Krysta H. Rogers, Stella McMillin, Katie J. Olstad, Robert H. Poppenga

**Affiliations:** ^1^ Wildlife Investigations Laboratory, California Department of Fish and Wildlife Rancho Cordova California USA; ^2^ California Animal Health and Food Safety Laboratory System, University of California, Davis Davis California USA

**Keywords:** American goldfinch, Drench application, Imidacloprid, Neonicotinoid, Songbird, Toxicity

## Abstract

In March 2017, 26 American goldfinches (*Spinus tristis*) were found dead following a drench application of imidacloprid in California (USA). Identical seed fragments were present in the digestive tracts. Imidacloprid was detected in 4 separate pooled samples from 18 birds, in crop/gizzard contents (4.8 ± 1.3 ppm; range 2.2–8.5 ppm) and liver tissues (3.9 ± 0.6 ppm; range 2.1–4.8 ppm). We suspect that fallen elm (*Ulmus* sp.) seeds were contaminated with imidacloprid during the drench application and subsequently ingested, resulting in acute toxicity and death. *Environ Toxicol Chem* 2019;38:1724–1727. © 2019 The Authors. *Environmental Toxicology and Chemistry* published by Wiley Periodicals, Inc. on behalf of SETAC.

## INTRODUCTION

Imidacloprid, a neonicotinoid insecticide, is synthetically derived from nicotine, a natural insecticide found in the leaves of tobacco and other plants (Jeschke et al. 2011). Introduced in the 1990s to help replace organophosphorous and carbamate insecticides, partly because of its relatively lower toxicity to humans and other vertebrates, imidacloprid has a broad range of applications worldwide for plant protection, structural and landscape pest control, domestic animal health, and aquaculture (Jeschke et al. 2011; Simon‐Delso et al. 2015). In California (USA) in 2016, imidacloprid was ranked 20th in pesticides used by cumulative acres treated (i.e., more than 1.5 million acres and approximately 64–000 applications); the applications were highest for structural pest control, landscape maintenance, animal husbandry, and crops such as grapes, lettuce, and cotton (California Department of Pesticide Regulation 2016). Imidacloprid is highly water soluble and is distributed systemically throughout all plant tissues, making it particularly effective against sucking, and some chewing, insect pests (Simon‐Delso et al. 2015). However, imidacloprid is also distributed into the plant's pollen and nectar (Stoner and Eitzer 2012), which exposes nontarget insects such as pollinating honeybees (*Apis mellifera*) and bumblebees (*Bombus terrestris*; Chauzat et al. 2006; Mullin et al. 2010; Laycock et al. 2012). Other applications including the treatment of seeds (which are then planted) and soil drench may contribute to the contamination of soil and surface water, further exposing other nontarget invertebrates (El‐Naggar and Zidan 2013; Johnson and Pettis 2014; Chagnon et al. 2015).

Imidacloprid is an agonist of the nicotinic acetylcholine receptors in the nervous system (Gervais et al. 2010). During exposure, imidacloprid binds to the receptor, resulting in a depolarizing blockade that leads to paralysis and death (Buckingham et al. 1997). In insects, the nicotinic receptors are present only within the central nervous system, whereas in vertebrates they are present at neuromuscular junctions and the central nervous system. Imidacloprid binds more readily to the insects’ nicotinic receptors compared with vertebrates, and thus are selectively toxic to insects (Gervais et al. 2010). However, imidacloprid is still considered moderately to highly toxic to birds and mammals, with exposure resulting in tremors, muscle weakness, and ataxia (Gervais et al. 2010; Eng et al. 2017; Millot et al. 2017). Incidents of poisoning in free‐ranging birds have been most frequently documented following ingestion of imidacloprid‐treated seeds that remain on the soil surface after sowing or spillage (Millot et al. 2017). Reported cases typically involve species of Galliformes and Columbiformes (Berny et al. 1999; Millot et al. 2017; Botha et al. 2018), which have a tendency to ingest large quantities of seeds during a single bout of feeding. However, only 5 treated corn seeds, containing approximately 1 mg of active ingredient each, would be required to cause a potentially lethal dose for a 390‐g gray partridge (*Perdix perdix*; Goulson 2013). By comparison, incidents of imidacloprid exposure in songbirds, which are expected to occur, are poorly documented (Mineau and Palmer 2013). In a laboratory study, white‐crowned sparrows (*Zonotrichia leucophrys*) weighing roughly 28 g were fed the equivalent of 4 treated canola seeds or less than one‐tenth of a corn seed, which resulted in sublethal effects including reduced body mass and disrupted migratory behavior (Eng et al. 2017). Population declines in several insectivorous songbirds have also been correlated to imidacloprid use presumably through the loss of insect prey rather than direct ingestion of seeds (Ertl et al. 2018; Hallmann et al. 2014).

In the present study, we describe songbird mortality following a drench application of imidacloprid to trees in a residential neighborhood, including the results of postmortem examinations and toxicant screening, and the likely route of exposure.

## MATERIALS AND METHODS

### Incident description

On 17 March 2017, the Stanislaus County Agricultural Commissioner's office reported to the California Department of Fish and Wildlife's Wildlife Investigations Laboratory (Rancho Cordova, CA) a mortality incident involving songbirds in Modesto (CA, USA) following a pesticide application by the City of Modesto. On the morning of 16 March 2017, a drench application of imidacloprid (Malice 75 WSP; Loveland Products) was made to the base of 76 trees along the streets in residential neighborhoods encompassing an area of approximately 12 km^2^. The pesticide was reportedly mixed in a tank according to package directions. On the evening of 16 March 2017, residents of the 200 block of Elmwood Avenue reported to the city the presence of dead birds in their front yards and on the street. Elmwood Avenue had the single highest concentration of trees treated by the drench application; 7 of the 25 treated trees were located on the 200 block.

### Postmortem examination and toxicant screening

In total, 26 carcasses were collected on 17 March 2017 and submitted on ice packs to the Wildlife Investigations Laboratory for mortality investigation; 22 of the 26 were determined to be in acceptable postmortem condition for diagnostic evaluation. The birds were identified as American goldfinches (*Spinus tristis*), a migratory songbird, and were arbitrarily labeled A to W. Six carcasses (A–F) were submitted to the California Animal Health and Food Safety Laboratory (Davis, CA) for postmortem examination including histopathology and toxicant screening. Samples of brain, skeletal muscle, peripheral nerves, trachea, lungs, liver, heart, kidneys, spleen, esophagus, proventriculus, ventriculus, and intestines were collected in 10 % buffered formalin, paraffin‐embedded, sectioned at 4 µm, and stained with hematoxylin and eosin for histologic examination using light microscopy. An oropharyngeal swab was collected for influenza A virus testing by polymerase chain reaction (PCR). Gizzard contents and liver tissue were collected from the 6 birds and pooled for imidacloprid and strychnine testing. A gross necropsy was performed on the remaining 16 carcasses (H–W) at the Wildlife Investigations Laboratory. Crop/gizzard contents and livers were collected from 12 birds (L–W) and submitted to CAFHS, where they were pooled into 3 groups of 4 for imidacloprid testing.

Crop/gizzard contents and liver tissue were extracted as previously described for imidacloprid (Filigenzi et al. 2011). Imidacloprid screening and quantitative analyses were performed on a Q Exactive high‐resolution liquid chromatography–mass spectrometry (LC–MS) system (Thermo Fisher Scientific). Crop/gizzard contents were extracted using 5 % ethanol with ethyl acetate, and strychnine screening was performed on an Agilent 5977 gas chromatography–mass spectrometry system (Agilent Technologies). Liver tissue was extracted into acetonitrile, and strychnine screening was performed on the Q Exactive LC‐MS system. Values reported are mean ± standard error of the mean (SEM).

## RESULTS

All birds were aged as adults (after hatch year) based on plumage and time of year (McGraw and Middleton 2017). Four birds were female and 12 were male; sex was not determined for the 6 birds submitted to California Animal Health and Food Safety Laboratory. All 22 birds were in good body condition with adequate adipose reserves and well‐developed pectoral muscles. Mean body mass was 12.3 ± 0.8 g (range 10.5–14.0 g; *n* = 16). Gross necropsy findings were similar among the birds and included varying amounts of blood present in the body cavity, lungs and trachea, and/or bone of the skull and brain; 2 had fractures of the sternum. Fragments of white‐colored seeds were present in the esophagus and/or gizzard of the 16 birds examined at the Wildlife Investigations Laboratory; gastrointestinal contents were not recorded for the birds examined at California Animal Health and Food Safety Laboratory. Histopathology for the 6 birds examined at California Animal Health and Food Safety Laboratory was generally unremarkable. One bird had multifocal random lymphohistiocytic and heterophilic hepatitis with small numbers of interstitial lymphocytes and plasma cells suggestive of bacterial septicemia, which was considered incidental because only one bird had such lesions. Influenza A virus was not detected by PCR on a pooled sample of oropharyngeal swabs. Imidacloprid was detected in 4 separate pooled samples of crop/gizzard contents (4.8 ± 1.3 ppm; range 2.2–8.5 ppm) and liver tissues (3.9 ± 0.6 ppm; range 2.1–4.8 ppm; Table 1). Strychnine was not detected in a pooled sample of gizzard contents or liver tissue.

**Table 1:** Level of imidacloprid measured in each of the 4 pooled samples of crop/gizzard contents and liver tissues from 18 American goldfinches (*Spinus tristis*) found dead following a drench application of imidacloprid insecticide in Modesto (CA, USA) on 16 March 2017

Bird ID	Crop/gizzard contents (ppm)	Liver tissue (ppm)
A, B, C, D, E, F	2.2^a^	
L, M, N, O	8.5	
P, R, T, V	4.5	
Q, S, U, W	3.8	
A, B, C, D, E, F		2.1^a^
L, O, R, T		4.3
M, P, S, W		4.8
N, Q, U, V		4.5

^a^ Respective sample split between imidacloprid and strychnine screenings.

## DISCUSSION

We describe a mortality event involving songbirds associated with a drench application of imidacloprid insecticide on trees in a residential neighborhood. Imidacloprid poisoning was identified as the cause of death for the American goldfinches collected at the application site. This was based on the presence of identical seed fragments in the birds’ digestive tracts, the detection of imidacloprid in the digestive contents and liver, and the spatiotemporal characteristics of the mortality event in association with a known pesticide application. Aside from evidence of mild trauma, no other significant findings were observed during postmortem examination, and strychnine was not detected in digestive contents or liver tissues.

Previous reports of imidacloprid‐related mortality in birds have involved granivorous species and the ingestion of imidacloprid‐treated seeds either during sowing or after accidental spills (Millot et al. 2017). The route of exposure for the birds examined in the present study is likely ingestion of natural seeds contaminated during the drench application. Elm (*Ulmus* sp.) seeds were observed on the ground under the trees treated with imidacloprid at the location where the dead goldfinches were collected. American goldfinches feed on the seeds of elm and other plants including grasses and flowers (McGraw and Middleton 2017). We suspect that the fallen elm seeds were contaminated with imidacloprid during the drench application and the birds then ingested the contaminated seeds, resulting in acute toxicity and death. Identical ingested seeds, resembling elm seeds, were present in the digestive tracts of the goldfinches examined, and imidacloprid was detected in 4 separate pooled samples of crop/gizzard contents and livers from 18 birds. The level of imidacloprid ranged between 2.2 and 8.5 ppm in the crop/gizzard contents and 2.1 and 4.8 in the liver tissues. The similar levels of imidacloprid in the crop/gizzard contents and livers of the goldfinches suggests that the imidacloprid was absorbed quickly after ingestion. In a toxicokinetic study involving Japanese quail (*Coturnix japonica*) fed treated seeds, imidacloprid was rapidly and extensively absorbed from the gastrointestinal tract into the blood and distributed to the liver, kidneys, muscle, and brain within 1 h post exposure (Bean et al. 2019). Seed fragments were still present in the crops of the goldfinches at time of death, indicating that death occurred rapidly after ingesting the seeds, a finding that also was observed in partridges and pigeons found moribund or dead following the ingestion of imidacloprid‐treated seeds in France (Berny et al. 1999; Millot et al. 2017). Imidacloprid was detected at concentrations of 0.9 to 1706.0 ppm and 0.4 to 286.7 ppm in digestive contents and 0.6 to 15.0 and 0.3 to 43.5 ppm in the liver for partridges and pigeons, respectively (Millot et al. 2017). Given the high individual variability of imidacloprid concentrations, Millot et al. (2017) suggested that a liver concentration of 1 ppm or higher for imidacloprid in birds provided strong support for diagnosis of intoxication when coupled with postmortem findings of an acute pathology. In addition, Eng et al. (2017) found that even 4.1 ppm of imidacloprid (10 % of the median lethal dose for house sparrows) was enough to cause signs of intoxication in white‐crowned sparrows and is equivalent to consuming approximately 4 treated canola seeds. Given that goldfinches are 2 to 3 times smaller than white‐crowned sparrows, an even smaller dose is likely to result in debilitation and death. Following intoxication, the affected goldfinches likely fell either from a perch or when attempting to fly, resulting in the observed trauma, observations that have been reported in other birds that have ingested imidacloprid‐treated seeds (Berny et al. 1999; Millot et al. 2017).

The mortality event investigated in the present study highlights a previously unidentified risk of drench application for imidacloprid. The pesticide label states that the product should be applied to the base of the tree and directly to the root zone. Seeds, insects, or other invertebrates consumed by birds and other animals may be present within that zone. If these food items were contaminated during the drench application, they would be highly toxic to animals when ingested. Due to the negative impact of imidacloprid on pollinators such as honeybees (Gill et al. 2012), the US Environmental Protection Agency now requires pesticide labels to state that the application of imidacloprid should not occur during blooming but rather prior to or well after bloom, to minimize exposure to pollinators (US Environmental Protection Agency 2017). Based on the present investigation, we would also recommend that drench applications not occur during seed drop, to minimize the risk of exposure to animals that consume fallen seeds. At a minimum, mitigation measures should be enacted to prevent animals from immediately accessing areas treated with a drench application.

Increased, localized mortality of small songbirds, such as finches and sparrows, are periodically reported to the Wildlife Investigations Laboratory. Rarely are these incidents investigated due to the difficulty of acquiring suitable carcasses for postmortem examination. Thus incidences of mortality due to the potential ingestion of seeds or invertebrates contaminated during drench or other applications of imidacloprid insecticides is likely underinvestigated, especially if the birds die in a location different from where the application occurred. Even if investigated, imidacloprid may not be on the list of differential diagnoses unless it is known that a pesticide application took place, because the seeds or invertebrates present in the digestive tract, although contaminated, would appear as a natural finding. Given the hazards associated with imidacloprid applications to nontarget invertebrates, vertebrates, and the environment, practices that favor integrated pest management over the prophylactic use of pesticides would help minimize the risk of exposure (Bueno et al. 2011).

## Data Availability

Data associated with this research can be requested from the corresponding author by emailing krysta.rogers@wildlife.ca.gov.
